# Children With Fragile X Syndrome Display a Switch Towards Fast Fibres in Their Recruitment Strategy During Gait

**DOI:** 10.1111/jir.13238

**Published:** 2025-04-08

**Authors:** Fabiola Spolaor, Federica Beghetti, Weronika Piatkowska, Annamaria Guiotto, Roberta Polli, Elisa Bettella, Valentina Liani, Elisa di Giorgio, Zimi Sawacha

**Affiliations:** ^1^ Department of Women's and Children's Health University of Padua Padua Italy; ^2^ Department of Information Engineering University of Padua Padua Italy; ^3^ Città della Speranza Fondazione Istituto di Ricerca Pediatrica Padua Italy; ^4^ Department of Developmental Psychology and Socialization University of Padua Padua Italy

**Keywords:** fragile X Syndrome, frequency content, gait analysis, muscles fibre, surface EMG

## Abstract

**Background:**

Fragile X Syndrome (FXS) is a genetic disorder caused by the lack of FMRP, a crucial protein for brain development and function. FMR1 mutations are categorized into premutation and full mutation (FXSFull), with somatic mosaicism (FXSMos) modulating the FXS phenotype. Recent studies identified muscle activity alterations during gait in FXS children. This study aims to explore the relationship between these muscle activity changes and motor fibre recruitment strategies during gait in FXS children.

**Methods:**

Fifty‐four FXS children and fourteen healthy controls participated in the study. Gait trials at self‐selected speeds were recorded using four synchronized cameras and a surface electromyography system that captured bilateral activity of Gastrocnemius lateralis, Tibialis anterior, Rectus and Biceps femoris muscles. The continuous wavelet transform, using the ‘bump’ mother wavelet, provided the percentage distribution of signal energy across nine frequency bands (50‐Hz increments within a 450‐ to 10‐Hz spectrum) and the Instantaneous MeaN Frequency (IMNF) time‐frequency distribution.

**Results:**

Results indicated that both FXSFull and FXSMos children exhibit a distinct fibre recruitment strategy compared to controls, with a higher percentage of total energy and elevated IMNF (*p* < 0.05).

**Conclusions:**

This increased reliance on fast‐twitch fibres may contribute to the observed fatigability and exercise intolerance in FXS children.

## 1 Background

Mutations of the *FMR1* gene, responsible for a spectrum of clinical problems ranging from neurodevelopmental to age‐related neurodegenerative conditions, are relatively common in the general population worldwide (Hagerman et al. 2018). Fragile X (FX) disorders, as a whole, are due to the effects of pathogenic expansion of a CGG trinucleotide‐repeat region located in the 5′ UTR of the FMR1 gene, on the X chromosome (Pretto et al. 2014). Individuals with *FMR1* full mutations ([FXSFull] > 200 repeated units associated with methylation of the entire region) are affected with FX Syndrome (FXS) due to transcriptional silencing with deficiency or absence of the protein FMRP, a protein essential to brain development and function. The classical symptoms of FXS are cognitive, with mild‐to‐moderate intellectual disability (Jacquemont et al. 2007; Rajaratnam et al. 2017), as well as behavioural, with anxiety, attention deficit, hyperactivity and social impairments with autistic features. Connective‐tissue anomalies, muscle hypotonia, facial dysmorphic features and macroorchidism in adult males, are also typical (Jacquemont et al. 2007). FXS is recognized as the most common single gene cause of autism spectrum disorder (ASD) with about 30% of FXS children diagnosed with ASD (Lieb‐Lundell 2016; O'Keeffe et al. 2019). Hypersensitivity to sensory stimuli and poor social skills are also typical. In FXS individuals, physical impairments with potential impact on the musculoskeletal system are low muscle tone, connective tissue alterations leading to hyperextensible joints, knock‐knees, flat feet, scoliosis, sustained fatigability and exercise intolerance (Kaub‐Wittemer et al. 2017). These musculoskeletal features justify a referral for physical therapy that can identify rehabilitation strategies related to balance and motor control (Hagerman and Hagerman 2021; Hagerman et al. 2017; Lieb‐Lundell 2016). Individuals with *FMR1* gene expansion between 55 and 200 repeats are at risk of developing premutation associated conditions due to the toxic effects of increased levels of FMR1 mRNA (Hagerman et al. 2017). Somatic mosaicism refers to two main different situations: size mosaicism (i.e., the presence of alleles with different numbers of CGG repeats) and methylation mosaicism (i.e., partially methylated full‐mutation alleles). Somatic mosaicism does represent a strong FXS phenotype modulator. To the best of the authors knowledge, there is very limited literature reporting evaluation of both motor pattern and musculoskeletal alterations in subjects with FXS (Sawacha et al. 2021); in this context, only one paper reported results about a gait analysis assessment. In the evaluated subjects, a consistently altered gait pattern, associated with abnormal muscle function assessed through surface electromyography (sEMG), was reported: a prolonged muscle activity duration and a higher frequency of activations during both preswing and swing phases of the gait cycle were found in all analysed muscles. Conventional sEMG analyses provide information about the intensity of muscle activity over time (Koenig et al. 2018); unfortunately, this type of analysis does not allow making any consideration about the role played by fibre types in muscle activity alterations. When activated alpha‐motoneurons and related fibre types are the object of investigation, the analysis of sEMG signals in the frequency domain is required. The small alpha‐motoneurons (Aminoff and Daroff 2014) and related slow type I fibres are responsible for the lower frequencies in the signal and the large alpha‐motoneurons and related fast type II fibres are responsible for the higher frequencies. Wavelet analysis of sEMG signals provides information related to signal frequency and allows distinguishing between activities of slow type I muscle fibres and fast type II fibres (Koenig et al. 2018). The aim of the present study was to investigate whether the alterations detected in the sEMG signals of FXS children during gait in the time domain were accompanied by alterations in muscle fibre recruitment strategy. For this purpose, CWT analysis was applied, because in healthy (Kilby and Gholam Hosseini 2004; Strazza et al. 2018), diabetic subjects (Sacco et al. 2014) and in children with cerebral palsy, this technique allowed to identify different motor strategy patterns during gait (Lauer et al. 2007; Romkes et al. 2006). To the best of our knowledge, spectral attributes of sEMG signal during gait have not been previously investigated in FXS.

## 2 Methods

### 2.1 Participants

Appropriate informed consent was obtained for each subject enrolled in the study for participation, scientific use of the data and publication (Local Ethic Committee, Università Azienda Ospedaliera di Padova, Trial No. 46039, submitted 29 July 2019). Fifty‐four children with FXS were evaluated in a routine clinical setting at the Department of Women's and Children's Health, University of Padua. Within the FXS group, 35 FXSFull children (BMI [kg/m^2^]18.9 ± 6.6; age [years] 10.2 ± 3.6) and 19 children with somatic mosaicism (FXSMos, BMI [kg/m^2^] 17.15 ± 6.6; age [years] 9.6 ± 3) were evaluated, together with 14 age and BMI matched (no statistically significant differences) controls with typical neurodevelopment (control subjects: CS, mean [± SD] age of 9.4 ± 2.3 years and BMI of 19 ± 3 kg/m^2^) evaluated at the BioMovLab (University of Padua). Statistical analysis was conducted using the Kruskal–Wallis test with chi‐squared correction, followed by post hoc Wilcoxon rank sum tests with false discovery rate adjustment. The analysis revealed that both FXS groups had significantly lower gait velocities compared to CS (FXSFull vs. CS *p* = 5.93 × 10^−13^; FXSMos vs. CS *p* = 5.63 × 10^−06^). Additionally, FXSFull demonstrated the lowest velocity, with a significant difference observed between FXSFull and FXSMos (FXSFull vs. FXSMos *p* = 0.0005). An overall summary of the space–time parameters results has been reported in Figure S1.

Subject numerosity was defined based on power analysis (Whitley and Ball 2002) carried on a previously published dataset (Sawacha et al. 2021) considering the value of the envelope peak as a variable; a number of 11 subjects was found to be sufficient for our analysis, and our sample consisted of 18 FXS children and 15 controls.

FXS subjects were enrolled according to the inclusion/exclusion criteria as follows (Sawacha et al. 2021):

- Molecularly documented full mutation of the *FMR1* gene with expansions of more than 200 CGG repeats and methylation of the promoter and repeated sequence; possible size and/or methylation mosaicism.
- Ability to walk independently.
- Absence of documented traumatic orthopaedic comorbidities affecting the lower limbs, occurred within 12 months from the beginning of the study.
- Absence of documented neurological disorders.

BMI‐ and age‐matched subjects with typical neurodevelopment were enrolled as controls for the study according to the following inclusion criteria:

- Ability to walk independently.
- Absence of documented lower limb injures within 12 months of the beginning of the study.
- Absence of documented neurological disorders.

Molecular analysis was performed as follows (Filipovic‐Sadic et al. 2010; Monaghan et al. 2013).

Genomic DNA (gDNA) was extracted from peripheral blood leukocytes (PBL) and flaking cells of the oral mucosa on an automated Maxwell 16 Blood DNA Purification System (Promega, Milan, Italy) and quantified by spectrophotometer with NanoDrop TM (ThermoFisher Scientific, Waltham, MA, United States). To identify the full range of FMR1 CGG repeat expansions and the presence of AGG interruptions, genomic DNA (40–60 ng) was amplified with Amplidex FMR1 PCR kit (Asuragen, Austin, TX, United States) according to the manufacturer's recommended protocol. All amplicons were analysed by capillary electrophoresis (ce) on a 3130xl Genetic Analyzer (Applied Biosystems, ThermoFisher Scientific, Waltham, MA, United States) equipped with POP‐7 polymer and a 36‐cm array with the following injection and run protocol: injection voltage, 2.5 kV; injection time, 20 s at 15 kV for 2400 s, including oven temperature of 60°C; buffer temperature, 35°C; prerun voltage, 15 kV; prerun time, 180 s; first readout time, 200 ms; second readout time, 200 ms; voltage number of steps, 20; voltage step interval, 15 s; voltage tolerance, 0.6 kV; current stability, 5 μA; ramp delay, 1 s; and data delay, 60 s. GeneMapper v 4.0 software with ROX 1000 size ladder (Asuragen, Austin, TX, United States) was used for automated sizing of *FMR1* gene‐specific peaks in conjunction with mobility correction factors. Samples were classified as follows: < 45 CGG repeats, normal; 45–55 CGG repeats, intermediate range; 55–200 CGG repeats, PM; and > 200 CGG repeats, FM according to American College of Medical Genetics guidelines. DNA samples were analysed for methylation status using the AmplideX FMR1 mPCR reagents (Asuragen) according to the manufacturer's recommended protocol. Briefly, 160‐ng DNA samples were premixed with two plasmids: a digestion control (DigCtrl) and PCR reference control (RefCtrl). This premix was separately aliquoted to a control or methylation‐sensitive digestion reaction. Capillary electrophoresis (ce) on a 3130xl Genetic Analyzer (Applied Biosystems, ThermoFisher Scientific, Waltham, MA, United States) were performed with the injection and run protocol reported for sizing analysis with only variation for injection voltage at 2.0 kV. All alleles were detected using FAM‐labelled primers, but only the proportion of the protected methylated allele was available for PCR using HEX‐labelled primers. Lack of methylation at either HpaII site resulted in digestion and thus no amplification. The percent methylation (%Me) for each peak was calculated as a ratio of peak heights between digested (HEX) and undigested samples (FAM), normalized to the CGG control amplicon peak height according to Equation (1)

(1) $$\%{\mathrm{Me}}_{i}=\frac{\frac{{\text{Peak}}_{i},\mathrm{HEX}}{\mathrm{Ref}\;\mathrm{HEX}}}{\frac{{\text{Peak}}_{i},\mathrm{FAM}}{\text{RefFAM}}}\times 100\%,$$

where *Peak*_*i*_,*HEX* is the signal height in the HEX channel corresponding to the methylated fraction of *Peak*_*i*_,*FAM* from the control digestion reaction and *Ref HEX* and *Ref FAM* to the peak heights of the PCR reference peak in the HEX and FAM channels, respectively. Alleles are reported as unmethylated (< 10%), partially methylated (10%–80%) and fully methylated (> 80%).

### 2.2 Instrumental Assessment

Video and sEMG data were simultaneously acquired through four synchronized cameras (GoPro Hero7, 1080 × 1920 pixel resolution, 60 fps) and two sEMG systems (FreeEmg, BTS, 1000 Hz, and Cometa Vicon, 2000 Hz) that collected the activity of the Tibialis anterior (TA), Gastrocnemius lateralis (GL), Rectus femoris (RF) and Biceps femoris (BF) bilaterally.

### 2.3 Data Processing

**FIGURE 1:**
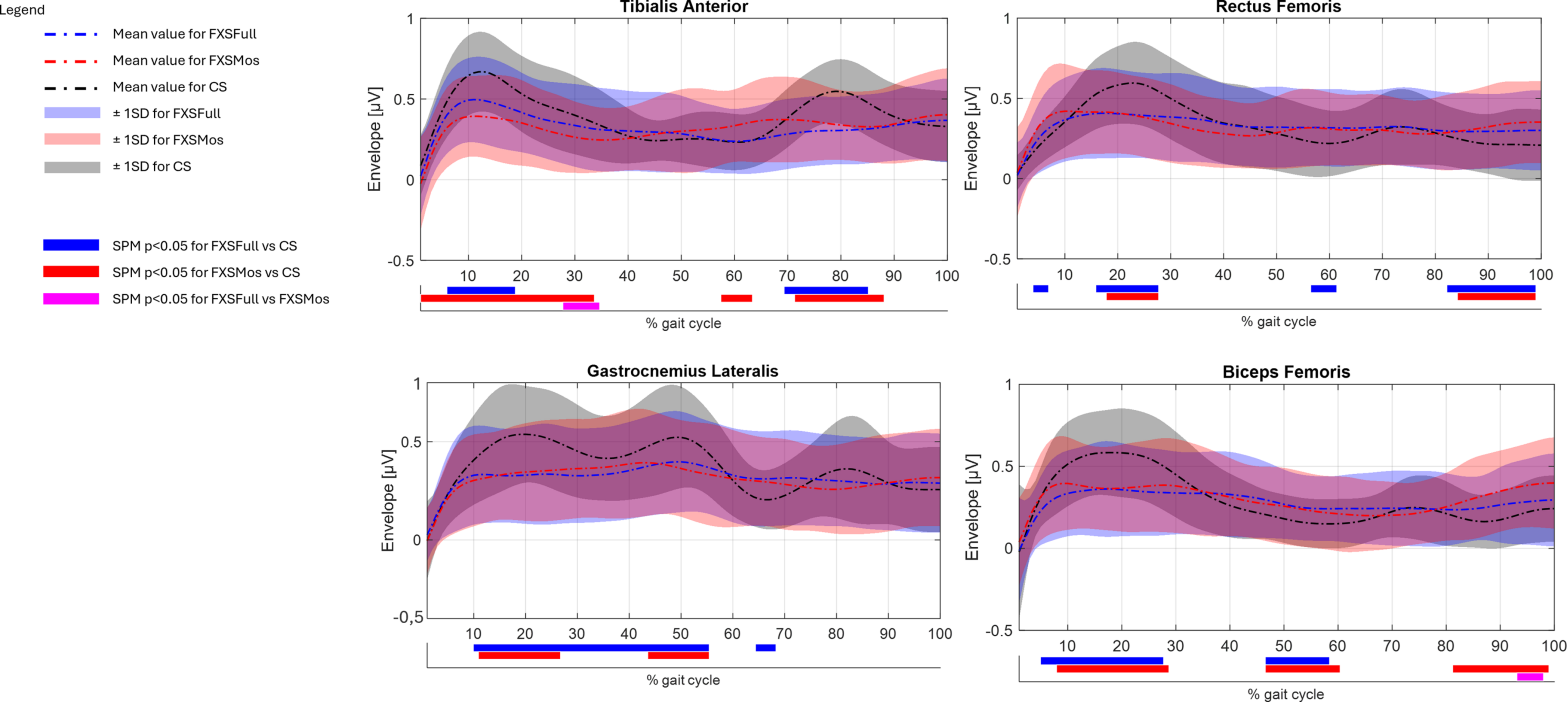
Mean of the envelope for FXSFull (blue dash‐dotted line), FXSMos (red dash‐dotted line) and CS (black dash‐dotted line) groups during the gait cycle for the Tibialis anterior (TA), Gastrocnemius lateralis (GL), Rectus femoris (RF) and Biceps femoris (BF). Standard deviation (± 1SD) of envelope for FXSFull (light blue–shaded area), FXSMos (light red–shaded area) and CS (light black–shaded area). Bars in the lower part represent the statistical significance (*p* < 0.05) using SPM: FXSFull versus CS in blue, FXSMos versus CS in red and FXSFull versus FXSMos in magenta.

Once acquired, the video sequences were processed through the software ‘Track on Field’ (BBSoF s.r.l.) in order to extract the spatiotemporal parameters as well as the gait cycle events for further sEMG processing purposes, as in Sawacha et al. (2021). All the sEMG signals have been interpolated and resampled, so signals with different frequencies of acquisition can be compared together. The sEMG signals were band‐pass filtered between 20 and 450 Hz using a double 5th‐order Butterworth filter, followed by full‐wave rectification, as described by Sawacha et al. (2012) to extract envelope profile (Figure 1). A double‐threshold statistical detector was employed to identify the onset and offset times of muscle activity, as it provides more accurate detection of these events compared to single‐threshold algorithms (Bonato et al. 1998). For each signal, an amplitude threshold (*θ*), numerosity threshold (*ρ*) and observation window length (*m*) were set. The values of *θ* and *ρ* were chosen to minimize false‐alarm probability and maximize the probability of detecting activation while considering specific background noise. The background noise, used to estimate the signal‐to‐noise ratio, was determined through a statistical algorithm that does not require prior knowledge of the signal (Agostini and Knaflitz 2012). The signals were digitized with a window length of 30 ms (*m* = 30 ms), a suitable time frame for analysing muscle activation during locomotion (Bogey et al. 1992; Di Nardo et al. 2013). If any sample within the window exceeded the threshold, it was classified as a muscle contraction and assigned a value of 1. Conversely, muscle relaxation was represented by a value of 0. A postprocessing step was applied to discard bursts shorter than 30 ms, with activation onsets defined as transitions from 0 to 1 and offsets from 1 to 0. Additionally, the digitized signals were used to generate activation maps as outlined by Benedetti (2012) (Figure S2). For further analysis, linear envelopes were calculated by applying a 4th‐order Butterworth low‐pass filter (5 Hz) to each signal, as described by Spolaor et al. (2021). For what concern time‐frequency analysis, the recorded sEMG signals were elaborated using CWT, which has the following general formula (Equation 2):

(2) $$W_{e}\left(a,b\right)=\int e\left(t\right)a^{\frac{1}{2}}\Psi \left(\frac{\underline{t-b}}{a}\right)\mathrm{dt},$$

where *a* is time, *b* is the scale parameter, *ѱ* is the mother wavelet and *e*(*t*) is an sEMG signal. *W*(*a*,*b*) represents the energy of the signal. In this study, the ‘bump’ mother wavelet was selected, described in terms of *σ* and *μ* by Equation (3):

(3) $$\hat{\Psi }\left(\mathrm{s\omega}\right)=e^{\left(1-\frac{1}{\frac{1-{\left(\mathrm{s\omega}-\mu \right)}^{2}}{\sigma^{2}}}\right)}1_{\left[\frac{\mu -\sigma }{s},\frac{\mu +\sigma }{s}\right]},$$

where *σ* defines the time/frequency localization. Smaller values of *σ* provide a wavelet with superior frequency localization but poorer time localization. The wavelet analysis was performed in Matlab (Version 2019a) using the command cwtft. The following frequency parameters were extracted: instantaneous mean frequency in each percentage of gait cycle and the percentage distribution of signal energy in frequency bands of fast and slow muscle fibres (450–125 Hz and 125–20 Hz) (von Tscharner 2000).

Instantaneous MeaN Frequency (IMNF) is an average frequency of power density spectrum of the signal for each percentage of gait cycle. It was calculated from time‐frequency distribution *W*(*f*,*t*) according to Equation (4):

(4) $$\text{IMNF}\left(t\right)=\frac{\sum_{j=1}^{M}f_{j}W\left(f_{j},t\right)}{\sum_{j=1}^{M}W\left(f_{j},t\right)}.$$

The time‐frequency distribution energy was computed for each band as a percentage of the total distribution energy according to Equation (5):

(5) $$E\left(\%\right)=100\times \frac{\sum_{f=f_{b}}^{f_{a}}W\left(f,t\right)}{\sum W\left(f,t\right)},$$

where *f*_*a*_ and *f*_*b*_ are the frequency limits for each band. To allow comparison of applied techniques elaborating sEMG signal in the time‐frequency domain, the activation timing analysis using double threshold algorithm proposed in Bonato et al. (1998) was estimated together with envelope as in Spolaor et al. (2021).

### 2.4 Statistical Analysis

The data were checked for normality of distribution using Lilliefors normality test in MATLAB (v. R2019a). Because the hypothesis that data have normal distribution was rejected, the nonparametric Kruskal–Wallis tests (two‐tailed *α* = 0.05) with the chi‐square correction for the numerosity of the groups, followed by post hoc Wilcoxon rank sum tests with False Discovery Rate in MATLAB (v. R2019a) was conducted. For the statistical analysis of the IMNF, we performed the one‐dimensional statistical parametric mapping (1‐D SPM) (Pataky 2011).

## 3 Results

**FIGURE 2:**
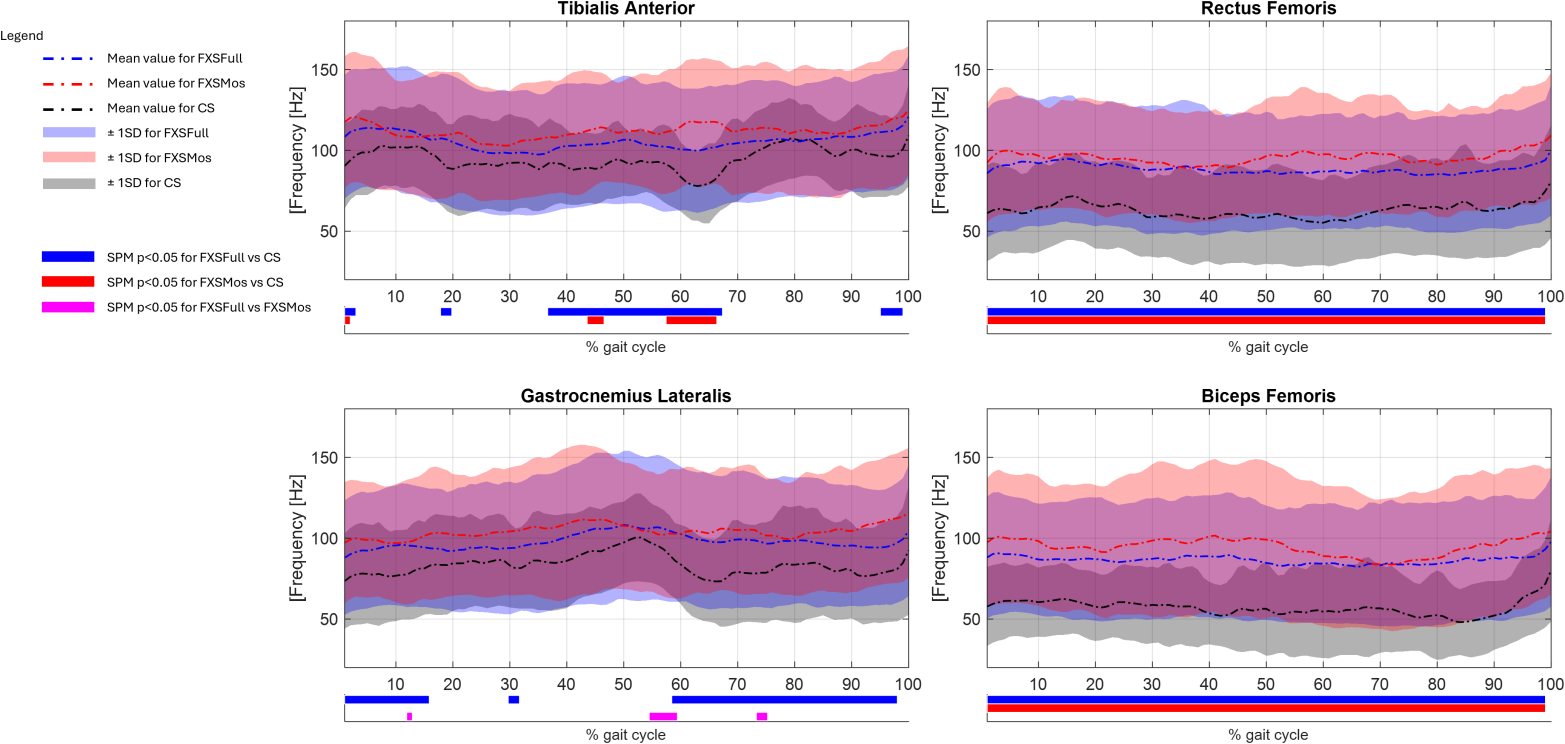
Mean of the instantaneous mean frequency for FXSFull (blue dash‐dotted line), FXSMos (red dash‐dotted line) and CS (black dash‐dotted line) groups during the gait cycle for the Tibialis anterior (TA), Gastrocnemius lateralis (GL), Rectus femoris (RF) and Biceps femoris (BF). Standard deviation (± 1SD) of IMNF for FXSFull (light blue–shaded area), FXSMos (light red–shaded area) and CS (light black–shaded area). Bars in the lower part represent the statistical significance (*p* < 0.05) using SPM: FXSFull versus CS in blue, FXSMos versus CS in red and FXSFull versus FXSMos in magenta.

**FIGURE 3:**
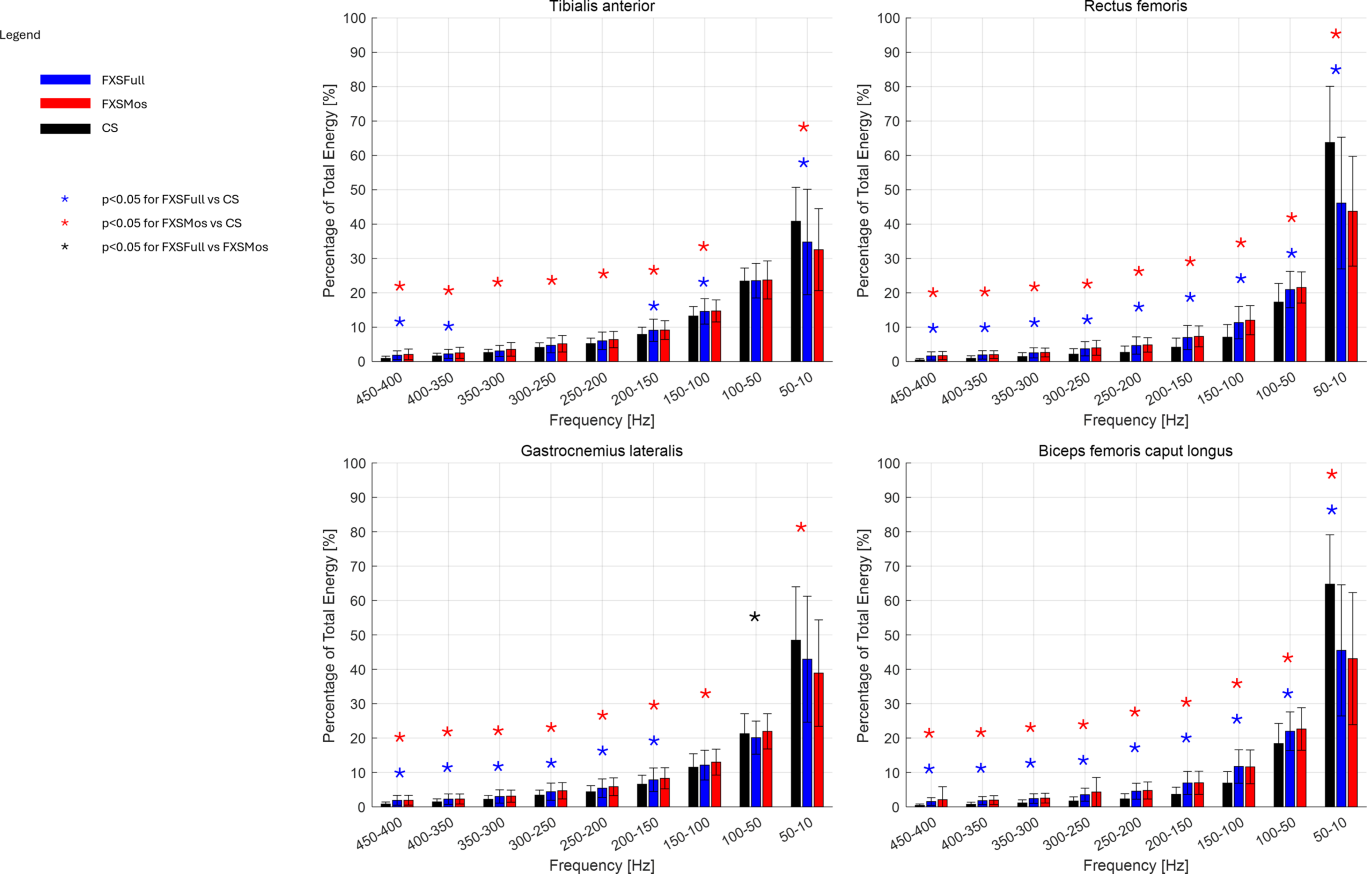
Energy content in the frequency nine band of 50 Hz in FXSFull (blue), FXSMos (red) and CS (black) groups for the Tibialis anterior (TA), Gastrocnemius lateralis (GL), Rectus femoris (RF) and Biceps femoris (BF). Median and interquartile range (IQR) with asterisk (*) when statistical differences are observed (*p* value of the Wilcoxon rank‐sum test < 0.05): blue FXSFull with respect to CS, red FXSMos with respect to CS and black FXSFull with respect to FXSMos.

**FIGURE 4:**
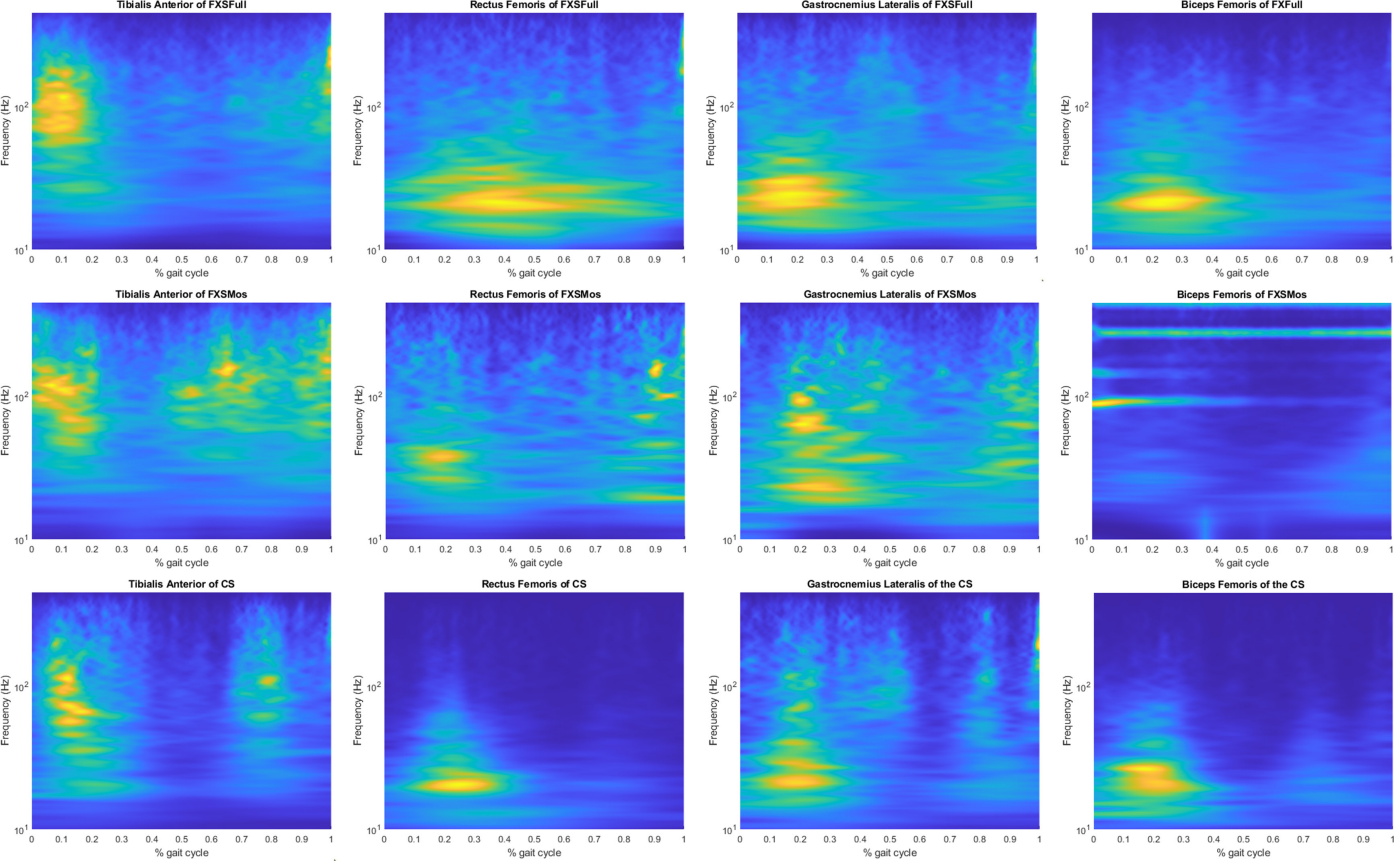
Average intensity pattern of FXSFull (in the top line), of FXSMos (in the middle line) and of CS (in the bottom line) for (from left to right) the Tibialis anterior (TA), Gastrocnemious lateralis (GL), Rectus femoris (RF) and Biceps femoris (BF) during the gait cycle (*x* axis), after wavelet analysis, in the frequency band considered 10–450 Hz (*y* axis).

Results of sEMG frequency analysis are reported in Figure 2 in terms of IMNF and in Figure 3 in terms of percentage of the total energy associated with the different fibre type. Results of sEMG analysis in the temporal domain are reported in Figure 4 as average of the envelope profiles for each subject cohort and in terms of on–off activity (Figure S2). In the same figures, the comparison among the three groups (CS, FXSFull, and FXSMos) for all these variables with respect to all the analysed muscles was also reported, along with statistical significance. Notably, no statistically significant differences were observed between left‐ and right‐side muscles with respect to the analysed variables; hence, the data were pulled together in the final analysis. Envelope profiles (Figure 1) revealed lower activation across all analysed muscles at initial contact, during loading response, and at initial stance, with the exception of the RF, which showed higher activation in FXSFull at initial contact. Both RF and BF exhibited increased activation during the swing phase, although for FXSFull, this difference was not statistically significant. The phase of the gait cycle where larger alterations were detected was the push‐off, which revealed statistically significant differences in all the analysed muscles in the comparison between the two FXS populations and CS; specifically, FXSMos displayed higher activation values in TA, whereas both FXSMos and FXSFull showed lower activation in the GL. In contrast, both FXSMos and FXSFull exhibited higher activation values in BF, and only FXSFull showed increased activation in RF during push‐off. IMNF profiles (Figure 2) presented increased values in both FXSFull and FXSMos groups with respect to CS for all the analysed muscles during the whole gait cycle:in details, when we take into account the results of the statistical analysis (see Section 2.4), RF and BF showed increased value for the entire gait cycle in both groups with respect to CS; TA showed higher IMNF in FXSFull during the terminal stance and preswing phases, whereas in FXSMos, higher IMNF was detected during the initial swing phase; considering GL, FXSFull showed increased values during the loading response and the swing phase. When comparing FXSFull with respect to FXMos, statistically significant higher values of IMNF were detected in the TA at the beginning of the midstance phase and in the GL in the preswing phase. The energy content in the frequency bands differed across all the analysed groups: Higher energy content was detected in both FXS groups with respect to CS in the energy bands associated with the fast twitch fibres, whereas lower energy content was observed in the energy bands associated with the slow twitch fibres. In particular, we detected a significantly higher energy content in TA in the highest frequency bands (450–100 Hz) in FXSMos, and in the following frequency bands in FXSFull: 450–400, 400–350, 200–150, and 150–100 Hz. For both FXS groups, a significantly lower energy content was recorded for TA in the lowest frequency band (50–10 Hz) with respect to CS. For GL, we detected a significantly increased energy content in the highest frequency bands (450–150 Hz) in both FXSFull and FXSMos with respect to CS; only FXSMos reported a significantly increased energy content with respect to CS in the 150‐ to 100‐Hz band. Both FXSFull and FXSMos showed statistically decreased energy content for the 100–50 to CS. Furthermore, FXSMos showed the lowest statistically significant energy content in GL with respect to FXSFull in the low frequency band (100–50 Hz). In comparing both FXSFull and FXSMos with respect to CS in terms of RF and BF, statistically higher energy content across the frequency bands 450–50 Hz was observed, whereas for the band 50–10 Hz, statistically significant lower frequencies were reported.

## 4 Discussion

The aim of this study was to explore whether the analysis of sEMG signals in the time‐frequency domain could offer valuable insights into the muscle fibre recruitment strategies in individuals with FXS. Our findings showed that the CWT analysis successfully identified the presence of differences between CS and FXS' neuromuscular control as a predominant recruitment of fast twitch fibres with respect to slow twitch ones was detected in FXS children, in general, during gait. Notably for some muscles (i.e., TA and GL), a significantly higher energy content was observed at all frequency bands between 450 and 100 in the FXSMos group with respect to CS, whereas in the FXSFull, this was not observed between 350 and 200 Hz in TA and between 150 and 100 Hz in GL. For what concerns the thigh muscles, similar alterations were observed in the energy content in both FXFull and FXSMos. Furthermore, we hypothesized that the presence of alterations in FXS muscle fibre recruitment strategy could explain the delayed and sometimes reduced sEMG activity (see Figure 1) detected through the standard sEMG analysis. Although our results did not support this hypothesis (Figure S3a–c), as no correlation was found between envelope peaks or duration of the activity and the energy content in the different frequency bands, the prevalent recruitment of nonfatigue resistant fibres in FXS could shed some light on other motor alterations commonly observed in FXS children such as fatigability and poor motor control (i.e., hyperactivity). Nevertheless, although FXMos displayed an activity closer to CS (i.e., TA and GL) in the analysis of the sEMG signal in the temporal domain, statistically significant differences were observed at the level of each frequency band with respect to CS in both FXS groups.

In the attempt to explain why FXS children display a different muscle fibre recruitment strategy, it is important to understand the damage a lack or reduction of the *FMR1* protein, FMRP, may play in the development and function of the brain tissue. FMRP acts as a negative regulator of mRNA translation for proteins involved in synaptic growth and maturation, and in the neuromuscular junction, the loss of FMRP stimulates the formation of supernumerary synapses. FMRP regulates the presynaptic release of Wnt ligands and the localization of postsynaptic receptors, influencing Wnt signalling transduction at the NMJ; again, its loss promotes excessive formation of synaptic boutons and increased synaptic transmission strength. Furthermore, the relationship between FMRP and neurons is crucial for balancing growth and synaptic maturation signals. Studies on the neuromuscular junction show that the loss of FMRP creates ‘hyperactivity’ in transsynaptic signalling, contributing to motor and cognitive dysfunctions. To go into further detail, FMRP contains several functional domains, including two Agenet/Tudor domains for binding DNA and other proteins, nuclear localization and export sequences, and multiple RNA binding domains, such as KH0, KH1, KH2 and the RGG box (Richter and Zhao 2021). In the brain, FMRP binds to over 1000 mRNAs involved in synaptogenesis, cell–cell communication, cytoskeleton and microtubule modulation, and metabolic regulation (Richter and Zhao 2021). FMRP also binds to mRNAs encoding transcription factors and epigenetic chromatin modulators (Richter and Zhao 2021). FMRP is also expressed in astrocytes where it regulates astrocyte‐neuron interactions; its deficiency may lead to transient impairment in white matter development (Richter and Zhao 2021). Furthermore, recent studies have described metabolic impairments in FXS, although the molecular mechanisms associating lack of FMRP to metabolic alterations are not clear yet (Abbasi et al. 2024). In the FXS children we have tested, energy content in frequency bands was increased in the higher frequencies (associated with fast twitch fibres) and reduced in low frequencies (associated with slow twitch fibres), thus indicating an alteration in muscle properties and MU recruitment strategies (Pilkar et al. 2017; Sacco et al. 2014; Wakeling 2009). It is interesting to note that previous works investigating diabetic subjects, with and without peripheral neuropathy, reported that alterations of the energy content detected in different frequencies could suggest changes in the proportion of recruited muscle fibre types (Pilkar et al. 2017). These subjects, independently from the presence of neuropathy, showed a reduced recruitment of type I muscle fibres associated with lower frequencies and an increased recruitment of type II fibres associated with higher frequencies (Lauer et al. 2007; Romkes et al. 2006). This strategy, which does not seem to be related to the neuropathy, was explained based on the metabolic, mitochondrial alteration of this disorder. Similarly, in FXS, an increased oxidative stress is present, which might be responsible for the reduced capacity of recruiting slow twitch motor fibres (i.e., low frequency content) particularly rich in mitochondria. In this hypothesis, the observed increased recruitment of fast twitch motor fibres (i.e., high frequency content) could represent a mere compensatory muscle mechanism. Although the idea of a metabolic basis for this muscular alteration in FXS is attractive, this might not be the only possible mechanism involved. Indeed, FXS is a neurodevelopmental disorder, characterized by altered brain development and function. An alternative hypothesis to the metabolic explanation would therefore be a functional neuronal disorder that could primarily impair muscle fibre recruitment by favouring the fast twitch ones. This second hypothesis could get along with the alterations our group previously detected, in the time domain, showing a higher frequency of muscle activations in FXS during preswing and swing phases in all analysed muscles. Additionally, FXSFull displayed a pattern of multiple short activations throughout the gait cycle. In terms of peak of the envelope, higher values, a delay in the position of the peak was highlighted in both FXSFull and FXSMos in all analysed muscles (Spolaor et al. 2021). Interestingly, the detected higher motor action potential amplitude (i.e., higher peak of the envelope), generally associated with larger motor units (Oberbach et al. 2006), supports the hypothesis of preferred use of fast twitch fibres in FXS individuals. Fatigue is a common and often debilitating symptom in individuals with fragile X‐associated conditions (FXPAC), including fragile X Syndrome (FXS) (Lieb‐Lundell 2016). Our data suggest that the altered muscle recruitment strategy observed in FXS may contribute to the higher fatigability reported by affected individuals, in addition to their existing hypotonia. This fatigue may be linked to underlying issues such as decreased mitochondrial function, which could represent a shared mechanism within the spectrum of motor dysfunctions seen in FXS and related disorders (Kaub‐Wittemer et al. 2017). Although FXS is a monogenic syndrome, FXS children share some common musculoskeletal alterations with other neurodevelopmental pathologies such as Dravet, Prader–Willi, Down and Williams syndromes. For example, in individuals with Down, Prader–Willi, Ehlers–Danlos and Williams syndromes, ligamentous laxity and hypotonia have been detected. Across these neurodevelopmental conditions, flat feet combined with joint laxity and hypotonia lead to increased activation of the Tibialis anterior and decreased activity of the Peroneus longus (Murley 2009). Variations in muscle activity among individuals with flat feet may reflect neuromuscular compensatory mechanisms aimed at reducing overload on the medial longitudinal arch (Murley 2009). Children with Dravet syndrome show motor control impairments; in particular, all children show delay above 2 years of age (Verheyen 2019). Moreover, motor neuropathy leads to crouching in these patients (Gitiaux et al. 2016). However, to the best of the authors' knowledge, no studies have reported an analysis of muscle fibre type in children with Dravet, Down, Ehlers–Danlos and Williams syndrome. Sone (1994) conducted a study on muscle fibres in Prader–Willi syndrome, highlighting the crucial role of muscle fibre type distribution in hypotonia and weakness. However, this study was based on histochemical and morphometric analysis, and no documented investigation through sEMG in dynamic conditions has been reported. This work comes with some limitations that should be acknowledged. First of all, the lack of data confirms these results at the level of muscle tissue. Muscle biopsies were not perceived as justified in FXS children and adolescents. By considering that translatability of the murine data to humans has been demonstrated in different previous studies, we plan to implement our data with analysis of tissue scanning from animal models (Willemsen and Kooy 2023). Also, a reduced muscle recruitment area should be mentioned, even though it is justified by the use of bipolar sEMG, because the behavioural characteristics of FXS children make the use of high density EMG impossible. The absence of speed control is also a limitation of the current study, as frequency analysis of sEMG is influenced by gait velocity, with faster speeds typically engaging fast‐twitch muscle fibres and slower speeds activating slow‐twitch fibres. This shift is observed in both healthy individuals and those with neurological conditions (Kamen and Knight 2004; Manca et al. 2017). However, in our cohort of children with FXS, controlling gait velocity is challenging due to motor coordination deficits and developmental delays. Unlike typically developing children, those with FXS exhibit irregular step lengths, slower speeds and reduced fluidity in movement (O'Keefe et al. 2016), making gait velocity difficult to regulate. Despite finding significant differences in gait velocities (see Table 1), our analysis did not show any correlation between gait speed and muscle fibre recruitment strategies (Figure S3a–c). Another limitation can be identified in the signal processing choice of adopting a bump mother wavelet analysis instead of the most frequently used Morlet and Daubechies (Strazza et al. 2018). This choice was justified by its characteristics that fit well with EMG‐signal: It is highly tunable and provides an optimal frequency resolution, and it is defined in the frequency domain (Nair et al. 2024). Bump mother wavelet has been used for analysing signal transitions and fast changes efficiently. Furthermore, the nature of this mother wavelet, which makes it frequency‐selective, helps in denoising the EMG signal. However, possible future developments could consider implementing different mother wavelets such as Morlet and Daubechies (Strazza et al. 2018), which were successfully applied in biomedical signal processing (Lee and Choi 2019). Finally, a limitation can be found in the lack of investigation about the association between blood‐based biomarkers and muscle health in the pathological cohort of subjects. However, given that medical procedures, including blood tests, can be challenging for ASD individuals and FXS in particular, due to heightened sensitivities and anxiety; in the present study, the introduction of further invasive tests, besides the ones strictly related to FXS diagnosis, was not considered. This in light of the fact that in children with FXS, no signs of a progressive degeneration of muscle function (i.e., muscle dystrophy, sarcopenia or neuropathy) have been documented, nor children in the present study underwent any sustained physical activity that could have justified investigating possible associations between sport performance and muscle function. However, as age‐related changes (between children and adults) in the muscular phenotype have been documented, although not yet fully characterized (Hagerman and Hagerman 2021; Haller et al. 2023; Jones et al. 2022), the association with blood related biomarkers could be explored in longitudinal studies, as a future development. Future developments will imply increasing the number of tested subjects and establishing a parallel study on FXS animal models complemented by muscle proteomic studies to confirm the identified muscle fibre type variation. Nevertheless, different signal processing analysis will be introduced to further confirm the current results. These findings, exploring for the first time the use of muscles in terms of recruitment strategy, can contribute to a better understanding of the motor control impairments associated with FXS. The relationship between the spectral attributes of sEMG signal and the changes in the recruitment of different muscle fibres should be further investigated both in humans and in animal models. If confirmed, these data could provide a further biomarker of the FXS motor control.

**TABLE 1:** Demographic data in terms of mean ± standard deviation for age, BMI and velocity.

Groups	No. of subjects	Age (years)	BMI (kg/m^2^)	Velocity (m/s)
FXSFull	35	10.2 ± 3.6	18.9 ± 6.6	0.85 ± 0.28
FXSMos	19	9.6 ± 3	17.15 ± 6.6	1.01 ± 0.28
CS	14	9.4 ± 2.3	19 ± 3	1.23 ± 0.19

## Conflicts of Interest

The authors declare no conflicts of interest.

## Supporting information


**Figure S1** Boxplot of the space–time parameters for CS (black dots), FXSFull (blue dots) FXSMos (red dots). Graph parentheses highlight statistical significance (*p* < 0.05) between groups.
**Figure S2** Frequency of muscle activation for the Tibialis Anterior (TA), Gastrocnemius Lateralis (GL), Rectus Femoris (RF) and Biceps Femoris (BF) during the gait cycle for FXSFull, FXSMos and CS. Horizontal bars are colour‐coded based on the number of subjects in which muscle activity is observed at each percentage of the gait cycle:yellow indicates muscle activity detected in all subjects, while dark green signifies no muscle activity detected in any subject.
**Figure S3** Spearman correlation for CS (a), FSXFull (b) and FXSMos (c) of the percentage of total energy for each band of frequency and velocity, peak of the envelope and first and second duration of activation for TA, GL, RF and BF. Red represents positive correlation while blue represents negative correlation.

## Data Availability

The data that support the findings of this study are available on request from the corresponding author. The data are not publicly available due to privacy or ethical restrictions.
