# Supplementary material for: Children With Fragile X Syndrome Display a Switch Towards Fast Fibres in Their Recruitment Strategy During Gait
Source: J Intellect Disabil Res. 2025 Apr 8;69(7):582–91. doi: 10.1111/jir.13238 (PMC12198096; doi:10.1111/jir.13238)
Supplement: Supplementary file 1 — Figure S1 Boxplot of the space–time parameters for CS (black dots), FXSFull (blue dots) FXSMos (red dots). Graph parentheses highlight statistical significance (p < 0.05) between groups. Figure S2 Frequency of muscle activation for the Tibialis Anterior (TA), Gastrocnemius Lateralis (GL), Rectus Femoris (RF) and Biceps Femoris (BF) during the gait cycle for FXSFull, FXSMos and CS. Horizontal bars are colour‐coded based on the number of subjects in which muscle activity is observed at each percentage of the gait cycle:yellow indicates muscle activity detected in all subjects, while dark green signifies no muscle activity detected in any subject. Figure S3 Spearman correlation for CS (a), FSXFull (b) and FXSMos (c) of the percentage of total energy for each band of frequency and velocity, peak of the envelope and first and second duration of activation for TA, GL, RF and BF. Red represents positive correlation while blue represents negative correlation. [file JIR-69-582-s001.docx]

**Supporting Information**


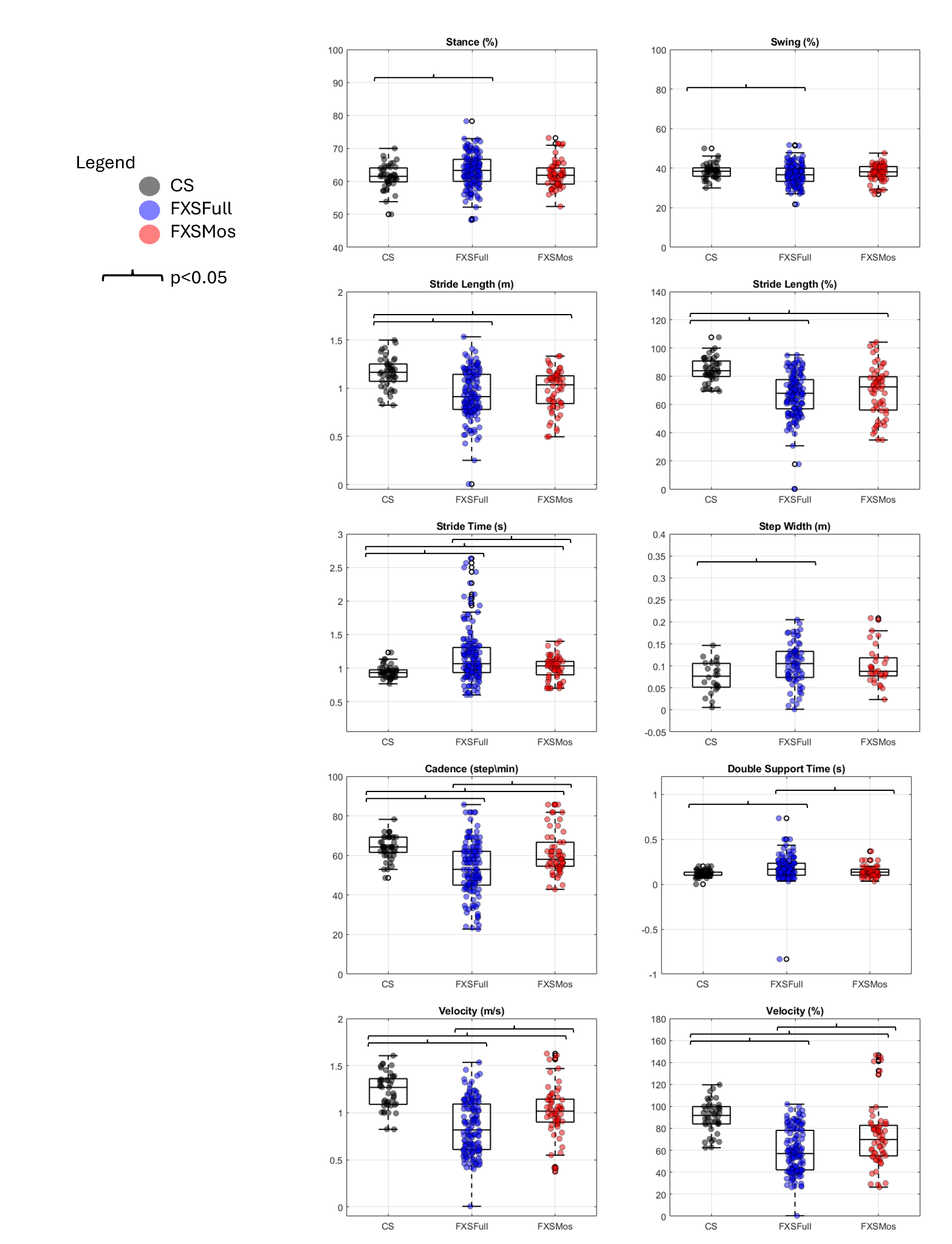


Figure_1_SuppInfo: Boxplot of the space-time parameters for CS (black dots), FXSFull (blue dots) FXSMos (red dots). Graph parentheses highlight statistical significance (p<0.05) between groups.


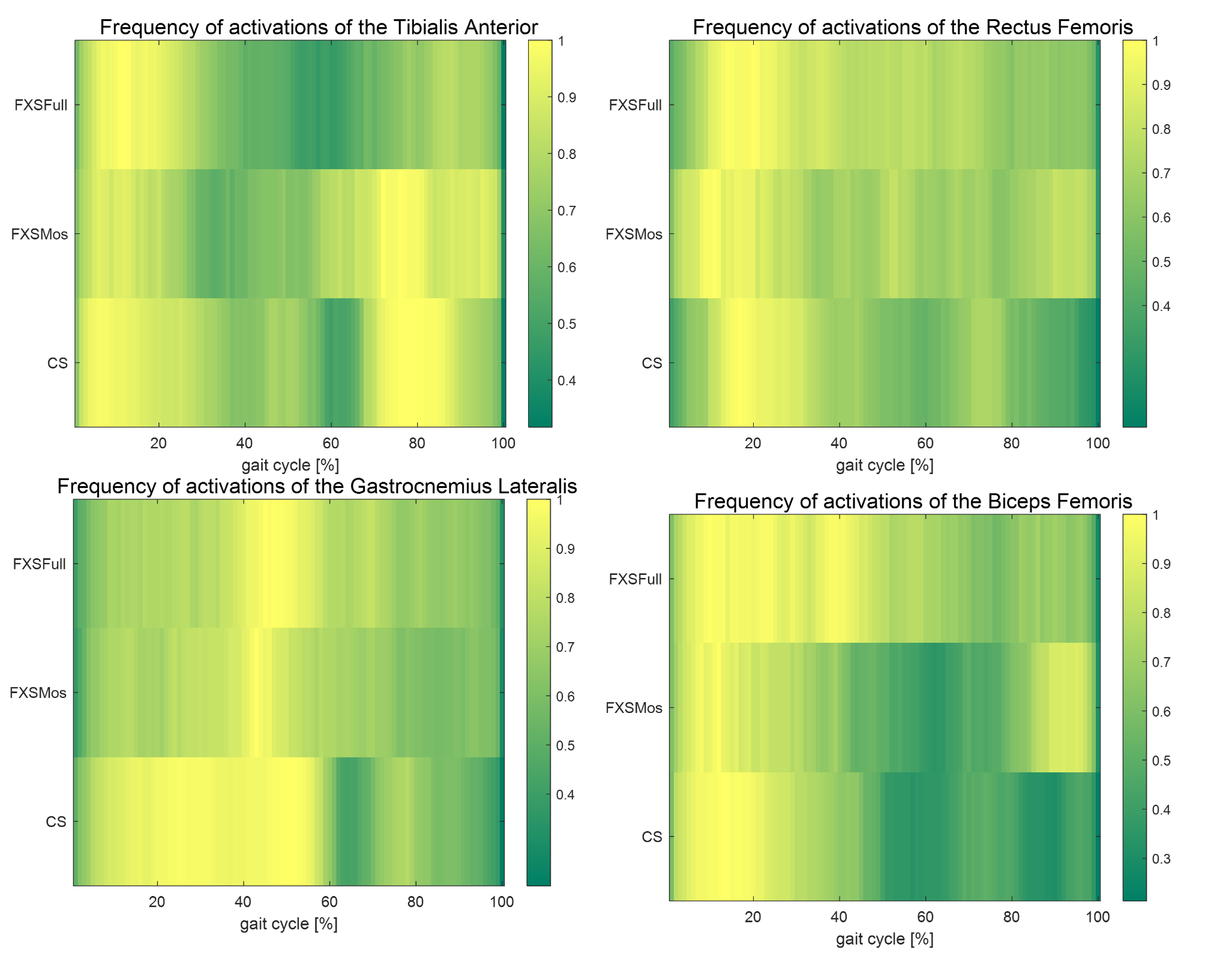


Figure_2_SuppInfo : Frequency of muscle activation for the Tibialis Anterior (TA), Gastrocnemius Lateralis (GL), Rectus Femoris (RF) and Biceps Femoris (BF) during the gait cycle for FXSFull, FXSMos and CS. Horizontal bars are color-coded based on the number of subjects in which muscle activity is observed at each percentage of the gait cycle: yellow indicates muscle activity detected in all subjects, while dark green signifies no muscle activity detected in any subject.


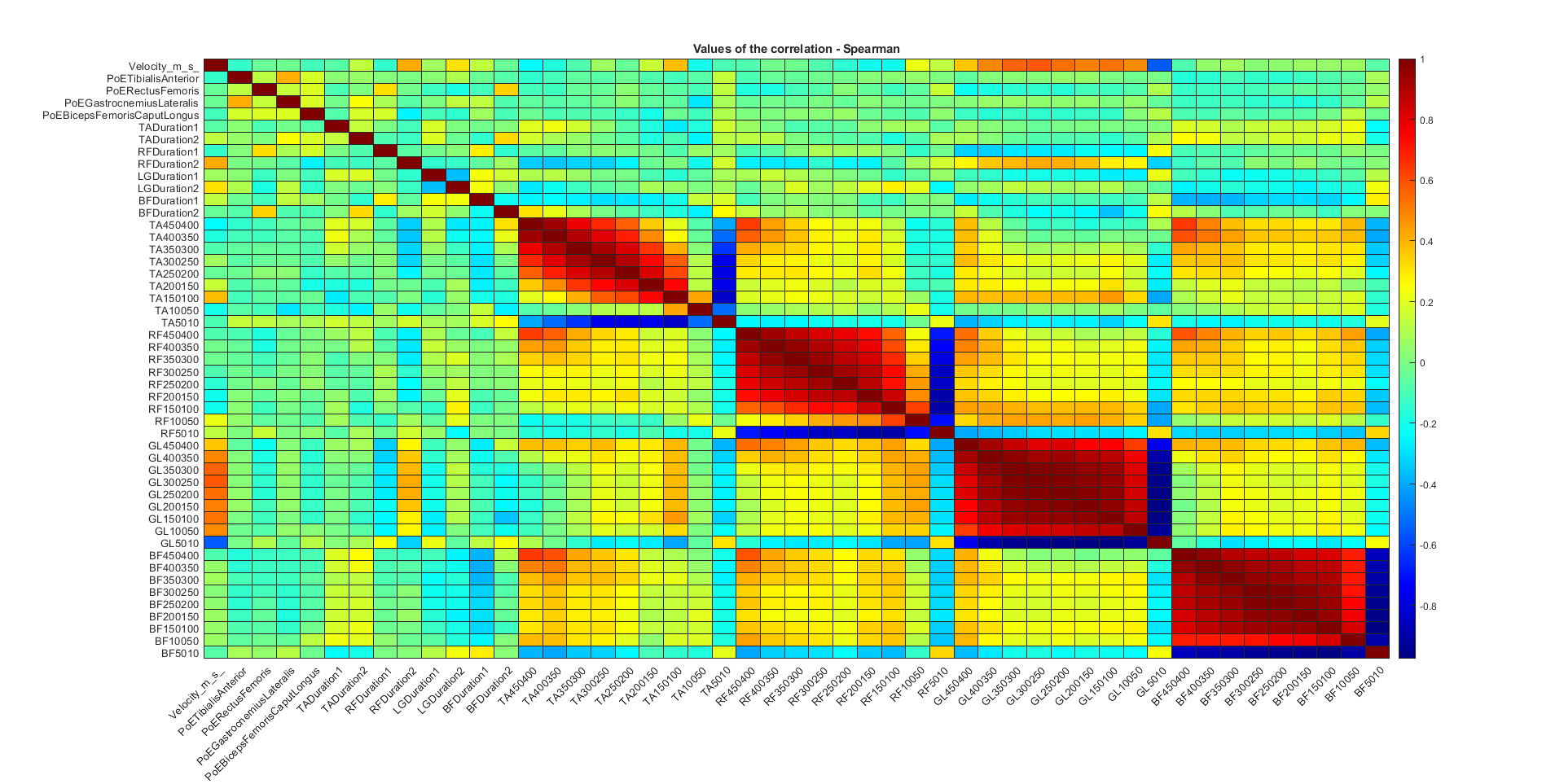

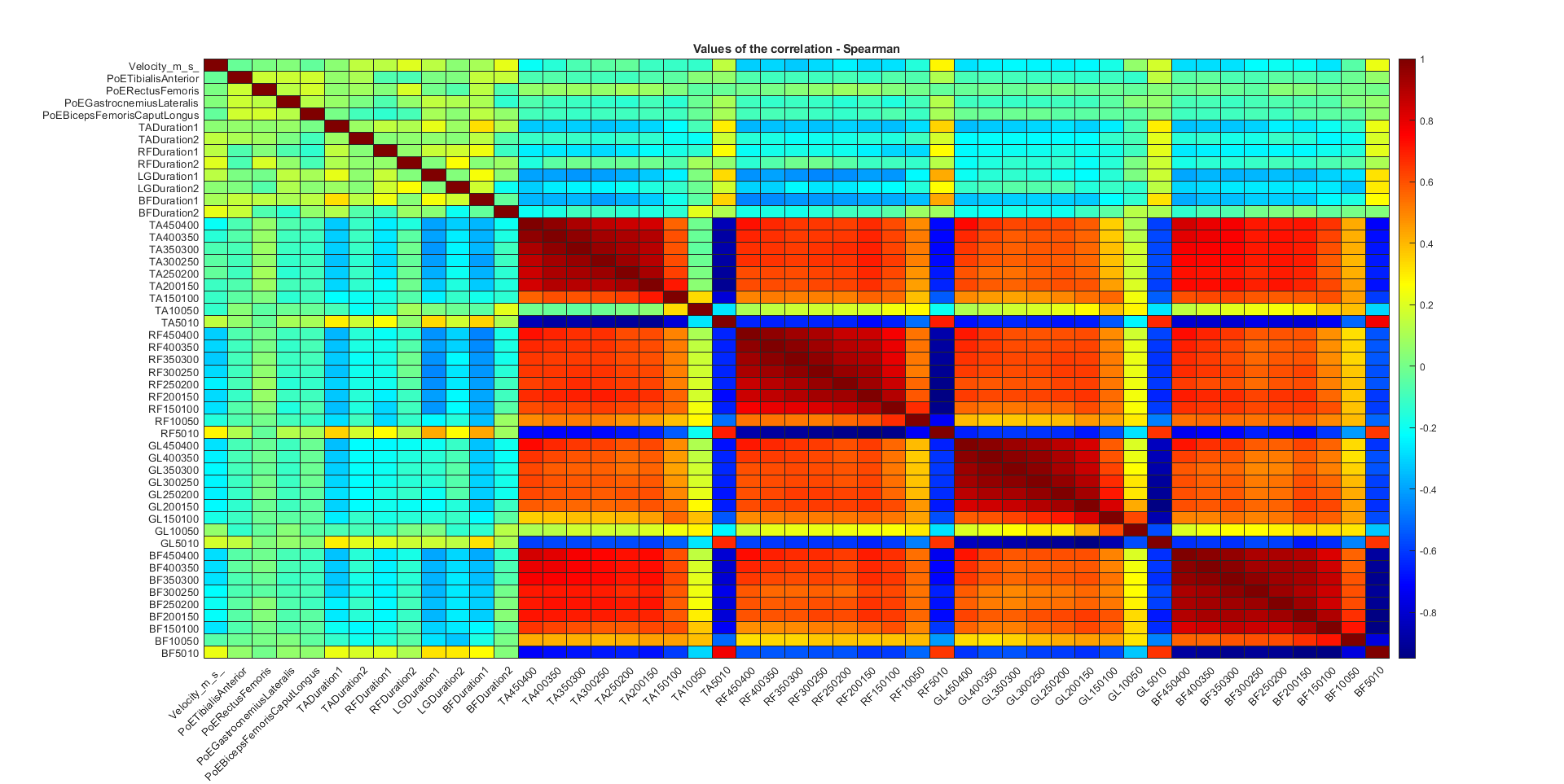

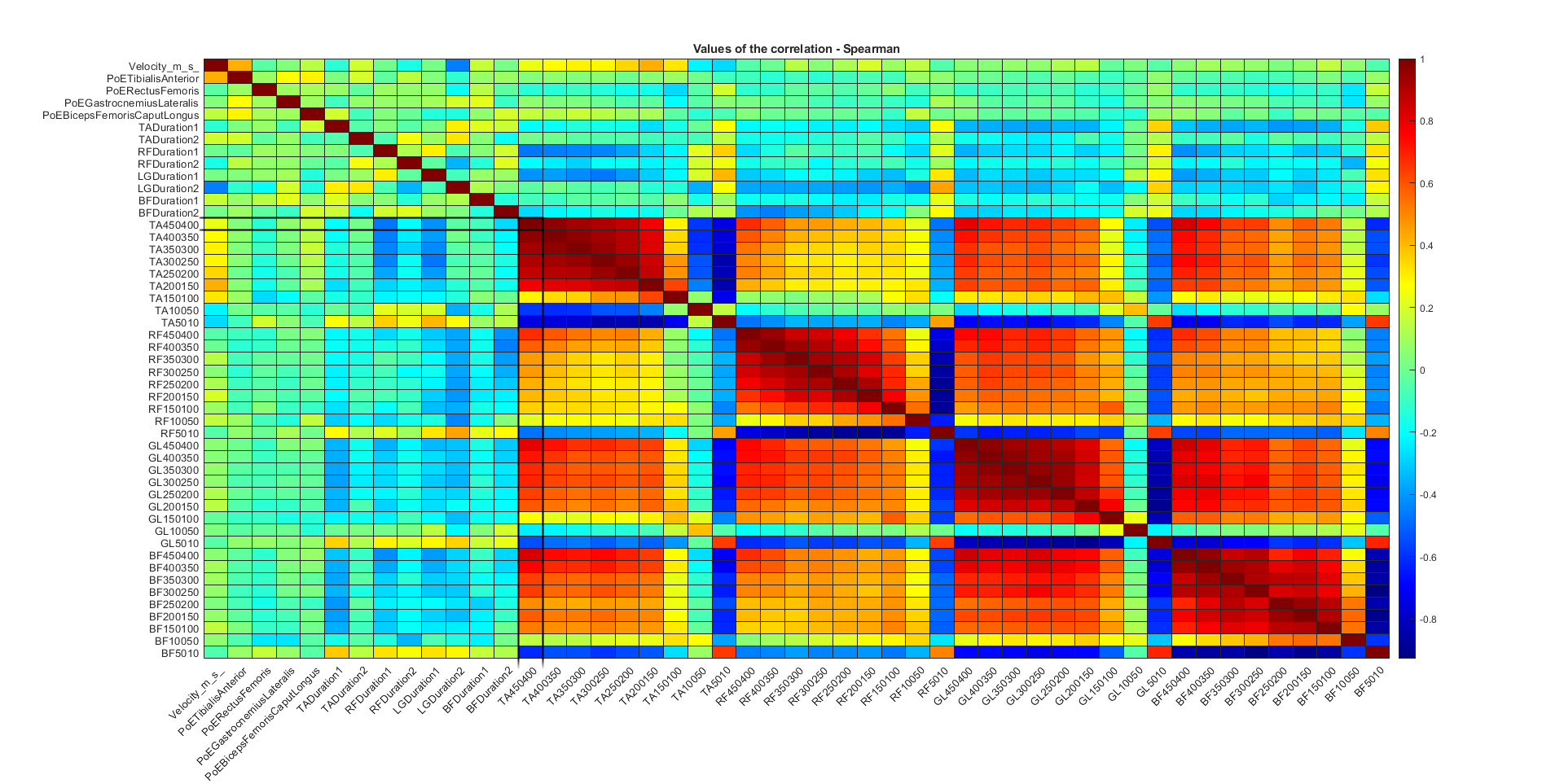


Figure_3_SuppInfo(a,b,c): Spearman correlation for CS (a), FSXFull (b) and FXSMos (c) of the percentage of total energy for each band of frequency and velocity, peak of the envelope and first and second duration of activation for TA, GL, RF and BF. Red represents positive correlation while blue represents negative correlation.
